# Current and Emerging Magnetic Resonance-Based Techniques for Breast Cancer

**DOI:** 10.3389/fmed.2020.00175

**Published:** 2020-05-12

**Authors:** Apekshya Chhetri, Xin Li, Joseph V. Rispoli

**Affiliations:** ^1^Magnetic Resonance Biomedical Engineering Laboratory, Weldon School of Biomedical Engineering, Purdue University, West Lafayette, IN, United States; ^2^Basic Medical Sciences, College of Veterinary Medicine, Purdue University, West Lafayette, IN, United States; ^3^Center for Cancer Research, Purdue University, West Lafayette, IN, United States; ^4^School of Electrical & Computer Engineering, Purdue University, West Lafayette, IN, United States

**Keywords:** breast cancer, magnetic resonance, MRI, diffusion, spectroscopy, contrast

## Abstract

Breast cancer is the most commonly diagnosed cancer among women worldwide, and early detection remains a principal factor for improved patient outcomes and reduced mortality. Clinically, magnetic resonance imaging (MRI) techniques are routinely used in determining benign and malignant tumor phenotypes and for monitoring treatment outcomes. Static MRI techniques enable superior structural contrast between adipose and fibroglandular tissues, while dynamic MRI techniques can elucidate functional characteristics of malignant tumors. The preferred clinical procedure—dynamic contrast-enhanced MRI—illuminates the hypervascularity of breast tumors through a gadolinium-based contrast agent; however, accumulation of the potentially toxic contrast agent remains a major limitation of the technique, propelling MRI research toward finding an alternative, noninvasive method. Three such techniques are magnetic resonance spectroscopy, chemical exchange saturation transfer, and non-contrast diffusion weighted imaging. These methods shed light on underlying chemical composition, provide snapshots of tissue metabolism, and more pronouncedly characterize microstructural heterogeneity. This review article outlines the present state of clinical MRI for breast cancer and examines several research techniques that demonstrate capacity for clinical translation. Ultimately, multi-parametric MRI—incorporating one or more of these emerging methods—presently holds the best potential to afford improved specificity and deliver excellent accuracy to clinics for the prediction, detection, and monitoring of breast cancer.

## Introduction

The American Cancer Society has estimated that within the United States in 2020, a total of 276,480 females will be diagnosed with breast cancer and 42,170 are likely to die from the disease (1). While breast cancer treatment has advanced, early detection remains a principal factor for improved patient outcomes and reduced mortality. Although, mammography has been the standard method of breast cancer screening since the 1960s, magnetic resonance (MR) imaging (MRI) offers superior sensitivity, particularly within denser breasts, and an annual MRI exam is recommended for high-risk women (e.g., women with familial history, genetic predisposition, significant chest radiation history, or lobular cancer) (2).

Amongst the existing and routinely practiced modalities to screen breast cancer, MRI has the highest sensitivity. In a recent study conducted over a period of eight years, Kuhl et al. reported a 95% confidence interval of 96.5–97.6% for specificity with a positive predictive value of 35.7% in diagnosing high grade breast tumors of sizes as small as 8 mm (3). A major limitation of clinical MRI lies in its wide range of specificity (37–97%) manifested as failures in differentiating malignant breast tumors vs benign lesions (4–6). However, false positive results from MRI observed in high risk lesions differ significantly from the low risk lesions associated false positive results through radiographs (7). These inherent biological differences with significant prognostic implications cannot be overlooked as we compare the results between MRI and other radiographic screening modalities. The advancements in MRI techniques and future research summarized in this paper are aimed at overcoming the specificity associated limitation of MRI to differentiate benign lesions from aggressive breast tumors with improved accuracy.

At present, secondary breast cancer prevention for males is not emphasized as widely as in females owing to the low male breast cancer incidence rate of 1% (8, 9). Studies demonstrating the use of MRI in screening male breast cancer patients are few, yet not uncommon (10–12). Survival outcomes of male breast cancer patients have worsened in recent years (12–14). The present treatment options for male breast cancer patients are derived from the clinical outcomes on female patients, which could be a potential limiting factor (14). Thus, more studies highlighting the impact of secondary breast cancer prevention on males, particularly given improved risk assessment from genetic testing, e.g., BRCA2-associated phenotype (15), are needed.

Advances in MRI and MR spectroscopy (MRS) have enabled clinicians to detect numerous biomarkers of breast cancer and to monitor the patient's response to chemotherapy. Studies have shown a correlation between these MR-based biomarkers and histopathological features of tumors. This linkage could provide a powerful technique for monitoring the progression of the disease and the patient's response to chemotherapy (16–21).

Image contrast based on tissue T_1_ and T_2_ are common MRI sequences exploiting the differences in the relaxation times of protons within the tissue under examination. T_1_ provides longitudinal relaxation time while T_2_ provides transverse relaxation time for a set of protons. By exploiting the distinct T_1_ and T_2_ relaxation properties of various tissues, static MRI provides superior structural contrast between adipose and fibroglandular tissues and remains a mainstay for risk analysis, tumor detection, and treatment monitoring. Dynamic MRI techniques go one step further, elucidating functional characteristics of malignant tumors. Dynamic contrast enhanced (DCE) MRI detects T_1_ changes in tissues over time immediately following bolus administration of a gadolinium-based contrast agent; the hypervascularity of breast tumors results in altered uptake and washout rates, and the unique time-intensity curve can distinguish malignant from benign tumors. Recent concerns regarding lasting gadolinium accumulation and toxicity, however, have impacted patient's assent to undergo techniques requiring gadolinium-based contrast agent, including DCE MRI, and research efforts have renewed to design alternative, noninvasive methods. One leading contender is diffusion weighted imaging (DWI), which already has proven valuable as an adjunct to DCE by improving combined sensitivity. DWI can elucidate tissue properties based on the Brownian motion of water. Since diffusivity differs inside and outside cells, the pattern of tissue morphology can be established based on the restriction of motion of water molecules in densely packed cells (22). Emerging techniques including MRS and chemical exchange saturation transfer can shed light on underlying chemical composition, providing snapshots of tissue metabolism and characterizing microstructural heterogeneity. Furthermore, non-compartmentalized, non-Gaussian diffusion models have the potential to derive micrometer-scale diffusion metrics that may reflect tumor heterogeneity and microstructural dimensions. This review article outlines the various MRI techniques currently used for breast cancer and examines several research techniques that demonstrate capacity for clinical translation or potential to facilitate discoveries in basic research.

## Current MR-Based Techniques

### Structural Imaging

**Figure 1:**
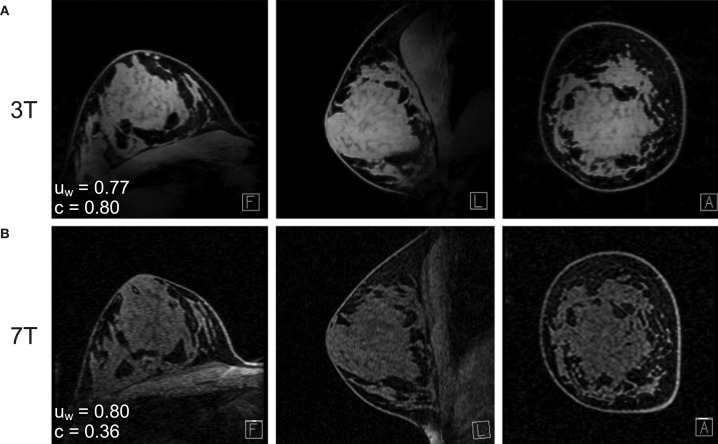
Fat-suppressed T_1_-weighted MRI of the same subject at **(A)** 7T and **(B)** 3T. The water signal uniformity (u_w_) is similar across 3T and 7T, while the fat-water contrast (c) is markedly improved at 7T. Reprinted with permission from Brown et al. (28); ©2013 Wiley Periodicals, Inc.

Among the clinical imaging modalities, MRI yields superior sensitivity of breast tumors and, notably among dense breasts, provides excellent contrast between tumor, adipose, and fibroglandular tissues (23, 24). A typical structural breast imaging protocol includes a T_2_-weighted sequence and a T_1_-weighted sequence, with and without fat suppression (25). Bilateral imaging is performed in order to evaluate asymmetries. High breast density is a known risk factor of developing malignant breast tumors (26), and specialized sequences have been developed for breast density measurement (27). The American College of Radiology Breast Imaging Reporting and Data System (BI-RADS) provides guidance for the succinct classification of overall breast composition, with emphasis on the proportion of fibroglandular tissues (25). As illustrated in Figure 1, fibroglandular tissues are readily differentiated from adipose tissues when using a T_1_-weighted sequence with fat suppression.

### Contrast-Enhanced Perfusion MRI

**Figure 2:**
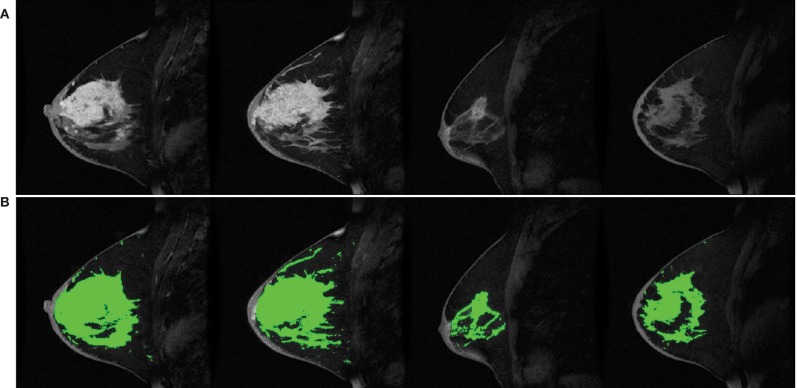
High-resolution 1.5T DCE MRI of four subjects from the American College of Radiology Imaging Network (ACRIN) 6657 repository (31) **(A)** unmodified and **(B)** with the segmented breast fibroglandular tissue overlaid in green.

Standard clinical breast MRI protocols also include a gadolinium dynamic contrast enhanced scan for distinguishing malignant from benign tumors. A fat-suppressed T_1_-weighted sequence is run before and up to 15 minutes after an intravenous bolus injection of gadolinium-based contrast agent followed by a saline flush. The rate of gadolinium washout is indicative of the microvascular properties and hyperintensity within malignant tumors is very sensitive and specific to malignant tumors (5). Notably, hormonal fluctuations can affect the uptake of gadolinium in healthy breast tissue, so dynamic contrast enhancement is only recommended to be performed during the first half of the menstrual cycle (29, 30). Representative dynamic contrast enhanced MRI are shown in Figure 2A.

In contrast to conventional dynamic contrast enhancement techniques, whole breast area (normal parenchymal breast tissues) can be enhanced utilizing the background parenchymal enhancement (BPE) technique. This technique can identify specific regions of differences within normal mammary tissues over others which facilitates a wider prediction of the tumor microenvironment and its possible changes. These features augment the specificity and sensitivity of MRI and is advantageous in reducing false positive results. BPE is assessed by four qualitative BI-RADS categories: minimal (<25% of glandular tissue demonstrating enhancement), mild (25-50% enhancement), moderate (50-75% enhancement), or marked (> 75% enhancement). In 2011, King et al. concluded that increased BPE is strongly predictive of breast cancer odds (32), however more recent studies have found no correlation with positive biopsy rate, sensitivity, or specificity (33).

### Clinical MR Scanners

Clinical 1.5 tesla (T) and 3T scanners typically include a built-in body coil for transmitting radiofrequency (RF) pulses, i.e., the B_1_ field. Given the off-center positioning of the breasts within the body coil, and the asymmetric loading presented by the torso, transmit B_1_ inhomogeneity is prone to worsen at higher magnetic fields. At 3T, the body coil has been reported to produce up to 50% error in tip angle (34), which significantly confounds the accuracy of quantitative image-derived measures including DCE enhancement ratio (35) and T_1_ mapping (36). These issues may be mitigated using advanced quantification techniques and accompanying pulse sequences, e.g., saturation-recovery snapshot-fast low angle shot (37).

Irrespective of the scanner's magnetic field strength, receive array coils improve signal-to-noise ratio (SNR) throughout the breast compared to utilizing the body coil to receive the RF signal (38). A variety of commercial breast receive array coils are available (39, 40) and custom 3T array coils have been reported to further improve performance for specific applications (41, 42).

## Emerging MR-Based Techniques

### Diffusion-Weighted MRI

#### Gaussian Models

##### Diffusion weighted imaging

**Figure 3:**
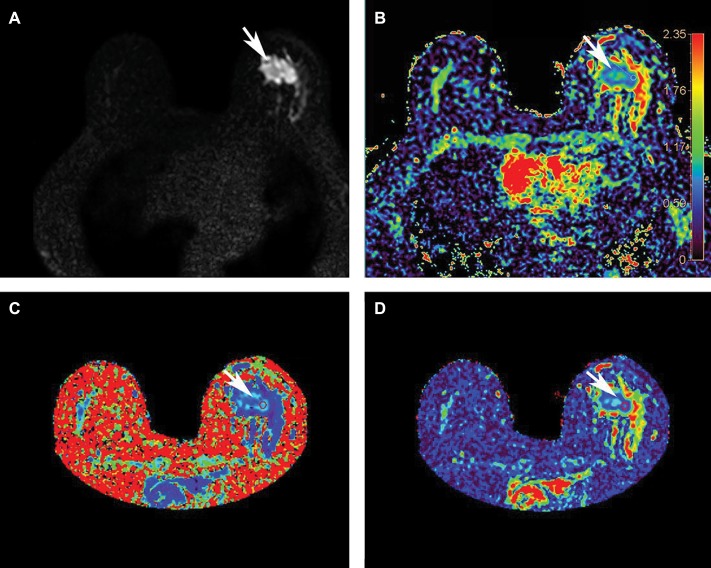
A comparison of diffusion techniques and metrics from scanning a 57-year-old woman with left breast invasive ductal carcinoma (tumor indicated by the arrow) at 3T. **(A)** The baseline b = 0 image acquired without diffusion gradients; **(B)** conventional DWI: apparent diffusion coefficient (ADC) map (scale bar 0-2.35 mm^2^/s), arrow indicating tumor ADC value of 1.090 mm^2^/s; **(C)** diffusion kurtosis imaging: mean kurtosis map (scale bar 0-3 mm^2^/s), arrow indicating tumor mean kurtosis value of 1.154 mm^2^/s; **(D)** DTI: mean diffusivity map (scale bar 0-2.8 mm^2^/s), arrow indicating tumor mean diffusivity value of 0.808 mm^2^/s. Reprinted with permission from Li et al. (43); ©2018 International Society for Magnetic Resonance in Medicine.

As a noninvasive MRI technique, diffusion weighted imaging (DWI) detects the bulk diffusion of water within tissue and offers substantial advantages in visualizing and differentiating tumors based on their vascularization patterns. The amount of diffusion weighting applied to the MRI signal is set by the operator-defined b-value, with zero indicating no diffusion weighting (Figure 3A) and commonly employed b-values for breast DWI being on the order of 1,000 s/mm^2^. DWI encodes water diffusion in one to three orthogonal directions (each direction corresponding to a gradient direction) and assumes unrestricted isotropic diffusion. The resulting apparent diffusion coefficient (ADC) quantifies the mean bulk diffusion per pixel and is an established quantitative surrogate for tissue cellularity. While the cell membranes and vascularity within tumors preclude unrestricted water motion, the simple DWI model accurately represents voxels (single data-specific locations on a 3D tissue construct) with high water content and low cell density and the resulting hypo intensity within breast tumors remains informative. This effect is illustrated in Figure 3B. Moreover, a technique known as automated DWI, which retrospectively computes higher b-value images from the typical DWI acquisitions, has been shown to improve lesion detection, particularly when calculations are performed on a voxel-wise basis (44).

Traditional spin-echo DWI relies on a conventional single-shot echo planar imaging readout prone to produce ghosting artifacts that hinder image quality. Other readouts such as spatio-temporal encoding mitigate ghosting artifacts at the expense of added noise (45). Ultimately, readout-segmented (or multi-shot) echo planar imaging has been established as a robust solution with good sensitivity; ghosting artifacts are prevented since each shot acquires the full extent of k-space in the phase-encode direction but only traverses a segment in the readout direction (46). The readout-segmented DWI sequence is prevalent and frequently prescribed for bilateral breast DWI with 2-mm in-plane resolution.

Higher-resolution DWI may be attained by reducing the field of view, which focuses on a target region within the breast. With this technique, 0.8-mm in-plane resolution can be resolved at 3T, and the resulting ADC maps provide greater detail facilitating the assessment of tumor morphology (47). Imaging time can be reduced by combining the high-resolution reduced field of view approach with multiband RF excitation (48).

Obtaining consistently high-quality breast DWI is one of the challenges that current studies are targeting to overcome. The American College of Radiology Imaging Network (ACRIN) 6698 clinical trial has shown that ADC can be measured with excellent repeatability and reproducibility in a multi-institution setting using a standardized protocol and QA procedure (49). An MRI platform that can provide a clearer distinction between tumors delivers more deterministic results to the patients, thus restricting the number of unnecessary biopsies performed on patients largely due to false positive results. However, it is important to note DWI should not be used as a stand-alone clinical protocol; rather, DWI hold a compelling role within multi-parametric MRI (mpMRI) protocols. For example, DWI detects significantly fewer cancers compared to dynamic contrast enhancement technique, but when incorporated as an adjunct it will yield superior sensitivity (46). Similar improvements can be achieved when pairing DWI with other complementary techniques such as MRS, as discussed later.

##### Diffusion tensor imaging

Diffusion tensor imaging (DTI) builds on the DWI technique by increasing the number of diffusion-encoding directions, thus enabling the calculation of anisotropic diffusion. While DWI characterizes isotropic diffusion within each voxel as a sphere, DTI employs at least six gradient directions and geometrically represents anisotropic diffusion within each voxel as an ellipsoid. The diffusion tensor, a matrix of directional diffusion coefficients, is established for each voxel based on the diffusion rates detected concurrent with each gradient configuration. Given the directionality of resulting diffusion information, DTI can provide additional insight into tissue microstructure through mean diffusivity—the DTI analogue to the ADC in DWI—and various anisotropy measures which provide critical information such as a tissue's vascularity, density, and cellular features. Such anisotropic features include fractional anisotropy, radial anisotropy, the individual diffusion coefficients, and the maximal anisotropy index. A mean diffusivity map is shown in Figure 3D.

While there is a consensus across studies that mean diffusivity is significantly lower in malignant tumors compared to benign lesions, there are conflicting results regarding the diagnostic utility of the anisotropy indices (50). Some reports suggest the standard DTI metrics of fractional anisotropy, radial anisotropy, and mean diffusivity cannot differentiate healthy tissue from cancer, while the diffusion coefficients and absolute maximal anisotropy index can assist in differentiating malignant tumors from both benign lesions and healthy tissue (51, 52). A recent approach suggests modifying the DTI model by compartmentalizing the diffusion signal as a combination of an anisotropic diffusion tensor (stroma cells) and a spectrum of highly-restricted (lymphocytes), restricted (cancer cells), and hindered (edema) isotropic-diffusion tensors; initial results with this modified diffusion basis spectrum imaging technique indicate greater diagnostic sensitivity and specificity distinguishing between malignant tumors and benign lesions (53).

Remarkably, DTI metrics have been shown to have distinctive correlations with breast cancer subtypes. Onaygil et al. found statistical significance between several anisotropy indices in estrogen receptor positive and negative (ER+ and ER-) breast cancers, and separate correlations with the levels of Ki-67, a biomarker for cellular proliferation, while Ozal et al. reported identifying distinct correlations between various DTI metrics and levels of breast cancer prognostic factors: ER, progesterone receptor (PR), human epidermal growth factor receptor 2 (HER2), Ki-67, and lymphatic invasion in invasive tumors (54, 55).

The challenge of achieving excellent repeatability and reproducibility across sites remains ongoing with breast DTI. Studies indicate the ADC can be reproduced with more accuracy compared to DTI anisotropy metrics such as fractional anisotropy (56, 57).

Notably, the technical development that drove substantial improvements into the DTI technique was largely motivated by the quest to map neuronal tracks of white matter in the brain. Preliminary studies reconsidering the utility of DTI for breast cancer have investigated utilizing DTI for breast tractography (58). Given the stark difference between the two-point connections of neuronal tracks and the branching ductal tree, Degani and colleagues proposed a novel computational methodology of post-processing DTI data using vector maps and clustering to infer the detailed structure of the mammary tree (59, 60).

#### Non-gaussian Models

##### Diffusion kurtosis imaging

While a Gaussian distribution of diffusion indeed applies to pure liquids and gels, barriers from complex tissue structures in effect modify the probability distribution of diffusion. Accordingly, the statistical metric for quantifying the actual probability distribution within tissue is designated as kurtosis. By acquiring additional, higher b-value images (where b value is an operator-defined parameter correlating with the strength and time for diffusion in imaged tissues), on the order of b = 1000–3000 s/mm^2^, and at least 15 diffusion gradient directions, the diffusion kurtosis imaging technique can map multiple structures within a single voxel, e.g., crossing white matter fibers in the brain. In the context of breast imaging, diffusion kurtosis imaging is sensitive to intracellular structures such as membranes and organelles (61) and, in addition to a mean kurtosis map, can provide a diffusion heterogeneity index sensitive to the tumor microstructure (62). Importantly, diffusion kurtosis analysis of the breast improves with correction for unsuppressed fat signal (63). A mean kurtosis map is shown in Figure 3C.

##### Intravoxel incoherent motion

While technically also a perfusion imaging method, the intravoxel incoherent motion model adds additional quantitative terms to account for microvascularity. Accordingly, intravoxel incoherent motion has the potential to discern both tissue diffusivity and microcapillary perfusion without the need for contrast agents (64). Additional quantitative metrics include the perfusion fraction (or blood volume fraction of vasculature) and a pseudodiffusion coefficient corresponding to water movement within microvasculature. For breast cancer imaging, the intravoxel incoherent motion model is more often added to non-Gaussian diffusion methods (65). A combination of high perfusion fraction, high kurtosis, and low diffusion coefficient is often observed at the periphery of tumors, while the opposite pattern is apparent in the necrotic core as well as within fibroadenomas (66). Accordingly, the intravoxel incoherent motion model shows promise for differentiating between malignant and benign breast lesions (67, 68). Furthermore, a recent report also indicates histogram analysis can accurately predict neoadjuvant chemotherapy (NAC) response (69).

#### Other Diffusion Models

Many other advanced diffusion methods have been proposed with the goal of probing intravoxel heterogeneity and cellularity; a review of several such methods and their suitability for cancer imaging was recently published by Tang and Zhou (62). Generally, these methods require additional acquisitions with b-values up to 4000 s/mm^2^, presenting a challenge given the lower SNR inherent with high b-value acquisition.

### Magnetic Resonance Spectroscopy

#### Proton Spectroscopy

Magnetic resonance spectroscopy (MRS) provides a localized snapshot of the biochemical makeup of tissue (70). Proton (^1^H) MRS offers the greatest sensitivity and simplest data acquisition. Elevated levels of choline-containing compounds indicate cell membrane turnover and are a biomarker for malignant breast tumors (71). All choline-containing compounds are quantified as total choline (tCho) and appear as a peak at 3.2 ppm on the ^1^H MRS spectrum. A thorough 2013 meta-analysis of tCho studies (*n* = 1193 patients) suggests this biomarker offers 73% sensitivity and 88% specificity (72). Moreover, high levels of glutathione measured with ^1^H MRS have been associated with increased resistance of cancer cells to radiation-induced cell death (73).

The recent ACRIN 6657 MRS clinical trial aimed to predict response to NAC with tCho single-voxel MRS; the results were inclusive, with only 29/119 subjects providing useable data (74). A primary limitation of the protocol was the manual placement of the MRS voxel within or encompassing the tumor, leading to issues with reproducibility across clinical sites. In the future this limitation can be addressed by running a full 3D magnetic resonance spectroscopic imaging sequence, allowing localized analysis to be performed retrospectively.

The high specificity of tCho studies suggests ^1^H MRS could be an effective addition to a mpMRI protocol (75). For superior differentiation of benign tumors from normal physiology, ADC values from DWI in combination with tCho peaks can provide a comprehensive result (76).

Proton MRS also facilitates lipid analysis, i.e., proportions of mono- and poly-unsaturated fats, fatty acid chain length, and mean saturation, all measures that are sensitive to past dietary intake. Specific lipid signatures have been reported to be significantly lower in malignant versus benign tumors, and luminal cancers can be differentiated via lipid MRS (77–79). Acquisition issues stemming from water-lipid susceptibility boundaries can be avoided by running a zero-quantum-coherence 2D MRS sequence (80).

#### Multinuclear Spectroscopy

**Figure 4:**
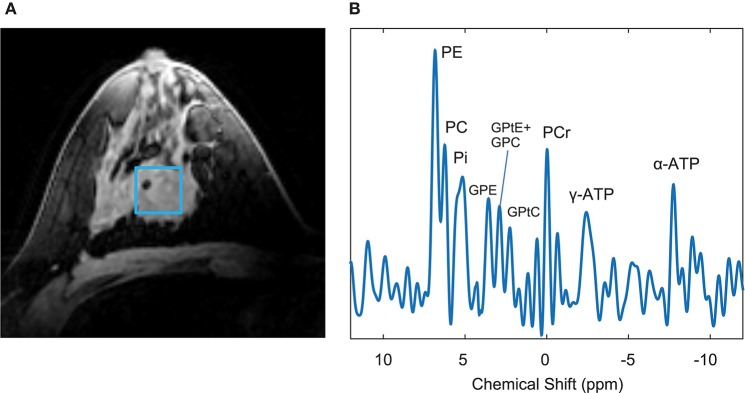
Example 7T data of a patient with an ER+, PR+, HER2- tumor. **(A)** T_1_-weighted image with indicated voxel selection (blue square), **(B)** ^31^P MRS spectrum of nine fitted metabolites. Adapted from Krikken et al. (88), used under CC BY.

With ^1^H MRS, many spectral peaks overlap and potentially mask lower-concentration metabolites. While multinuclear MRS suffers upfront from reduced sensitivity—an inherent deficit in SNR that is somewhat mitigated at higher fields—they provide a window into breast cancer metabolism with information inaccessible to ^1^H MRS (81). Phosphorus-31 (^31^P) MRS separates distinct choline compounds, specifically phosphorylcholine and glycerophosphocholine, otherwise overlapped as tCho on the ^1^H spectrum. The role of phosphocholines in breast cancer metabolism is of broad interest (82–85), with the ratio of phosphocholine to glycerophosphocholine hypothesized to switch from low to high during malignant transformation (86), and to increase further with tumor progression (87). The ratio of phosphomonoesters to phosphodiesters has been shown to decrease after successful NAC (88). An example ^31^P spectrum from an ER+, PR+, HER2- tumor is presented in Figure 4.

Carbon-13 (^13^C) MRS can provide additional information such as the composition of breast fat and correlations that may predispose to cancer. Performing *in vivo* ^13^C MRS is difficult for many reasons, including low natural abundance, low (in comparison to ^1^H) sensitivity, J-coupling bonds between ^1^H and ^13^C atoms that obfuscate spectral peaks, and unique hardware instrumentation requirements. The preferred ^13^C MRS experiment, applying broadband proton decoupling, requires RF coils operating at both the ^1^H and ^13^C frequencies; the ^1^H channel is used for scout imaging as well as to transmit proton-decoupling pulses across the J-coupled chemical shift band (89). By employing proton decoupling at 7T, natural abundance ^13^C lipid analysis from the breast was demonstrated (90). Enriched or hyperpolarized ^13^C studies boost the SNR and facilitate additional studies, including using ^13^C-labeled choline to distinguish between catabolic and anabolic pathways in choline metabolism (91), and gauging glucose metabolism in the breast using [U-^13^C] glucose bolus injection (92).

#### Magnetization Transfer

**Figure 5:**
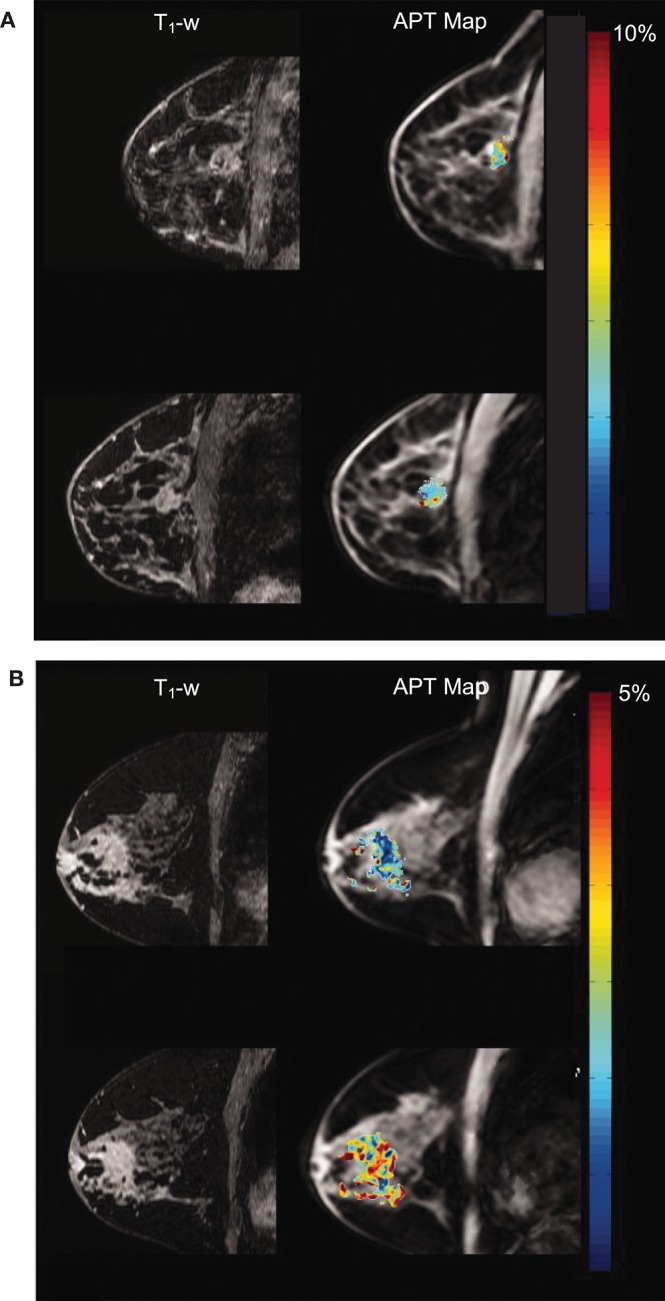
Amide proton transfer maps overlaying anatomical T_1_-weighted images acquired at 3T. The top row shows data acquired prior to neoadjuvant chemotherapy (NAC); the bottom row shows data acquired after one cycle of NAC. **(A)** Patient who had complete response (i.e., no residual tumor) and **(B)** patient who had progressive disease. Reprinted with permission from Chan et al. (95); ©2012 Wiley Periodicals, Inc.

Magnetization transfer (MT) was first introduced by Wolff and Balaban (93); the MT image contrast reflects the exchange of magnetization between protons in free water and protons bound to macromolecules due to chemical exchange and dipole-dipole interactions. After image acquisition with a specialized off-resonance RF pulse, the MT effect among voxels of interest is quantified using either the so-called z-spectrum or a histogram of the MT ratio. The repeatability of quantitative breast MT measurements among cohorts of healthy volunteers has recently been demonstrated (94, 95). MT images can provide important information of tumor response to NAC (96). Chemical exchange saturation transfer extends the capabilities of MRS by indirectly detecting low-concentration chemicals through their proton exchange with water, including protein aggregates in malignant tumors. For example, amide proton transfer imaging detects the protein and peptide concentration by saturating the amide protons within peptide bonds. Dula et al. defined an integrated voxel-wise metric assumed to reflect the cellular protein and peptide content, designated amide proton transfer residual, and calculated this measure before and after neoadjuvant chemotherapy for two women with ER- breast cancer who experienced contradictory outcomes (95). As illustrated in Figure 5, they found a decrease in amide proton transfer residual from the woman with a complete response, while the metric from the woman with progressive response increased (95). Moreover, chemical exchange saturation transfer can discriminate between nonmalignant and aggressive human breast cancer cells, as it can characterize the metabolites altered by breast cancer cell aggressiveness and chemotherapy response (97). For example, the amide proton transfer signal in triple negative tumors is distinct and may result from the unique microenvironment of the tumor subtype (98). In addition, amide proton transfer asymmetry is observed in patients with breast cancer treatment-related lymphedema (99). Notably, high quality amide proton transfer images can be readily obtained at 7T, because both the chemical exchange saturation transfer effect and SNR are enhanced at higher field strengths (100).

### Other Techniques

#### Sodium MRI

Sodium (^23^Na) is abundant in the body and, unlike other non-proton nuclei that yield spectra for chemical quantification, sodium has no chemical shift dispersion and instead produces images (101). Malignant tumors are thought to increase sodium content due to disruption of the sodium-potassium pump in cell membranes. Elevated tissue sodium concentration has been confirmed in malignant lesions (102), and sodium concentration correlates well with the ADC of DWI (103).

#### Susceptibility Weighted Imaging

**Figure 6:**
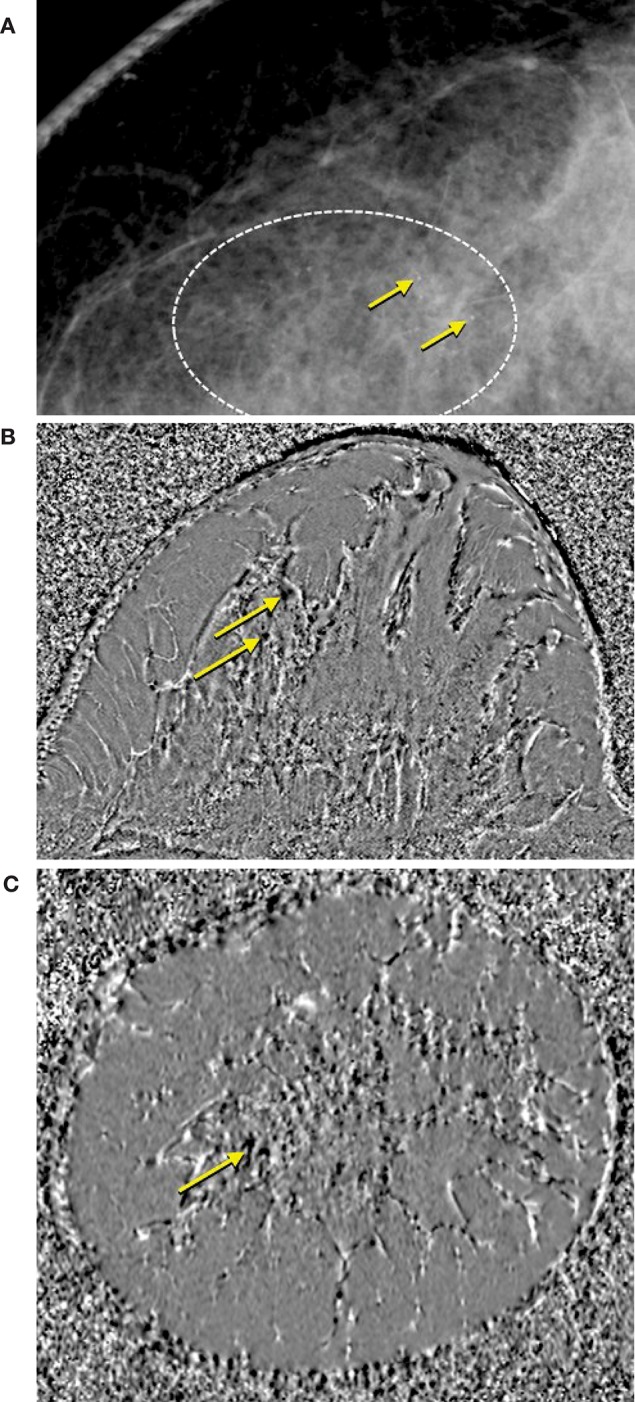
Comparison of **(A)** mammogram and **(B,C)** susceptibility weighted phase images acquired at 7T with a 0.35-mm isotropic resolution ${\text{T}}_{2}^{*}$-weighted 3D gradient echo sequence (105). Diamagnetic microcalcifications are indicated by yellow arrows and are hypointense in the susceptibility weighted phase images.

Historically recognized as the cause of frequent MRI artifacts, particularly near air-tissue interfaces or in the vicinity of metal implants, differences in magnetic susceptibility can also produce contrast between diamagnetic and paramagnetic tissues. Ductal carcinoma *in situ* (DCIS) is frequently missed by DCE MRI and has been shown to associate with certain patterns of breast calcifications (104). Calcium is more diamagnetic than tissue water, and the susceptibility effects are intensified at higher magnetic fields. Figure 6 illustrates the ability of 7T susceptibility-weighted MRI to identify microcalcifications otherwise only visible using mammography (105).

#### MR Elastography

MR elastography (MRE) images a low-frequency acoustic wave as it propagates throughout tissue. By calculating the local complex sheer modulus, MRE can characterize biomechanical properties of breast tissue including differences in stiffness. The initial aim of employing MRE for breast cancer was to differentiate benign lesions from malignant tumors; the more liquid-like behavior of malignant tumors provided sufficient MRE contrast to achieve this aim (106). More recently, MRE is being combined with 3D strain imaging, the latter altering the stress-load relation of tumors; ongoing studies are investigating the potential of MRE to determine mechanical forces to estimate the metastatic potential of tumors (107).

#### MR Fingerprinting

**Figure 7:**
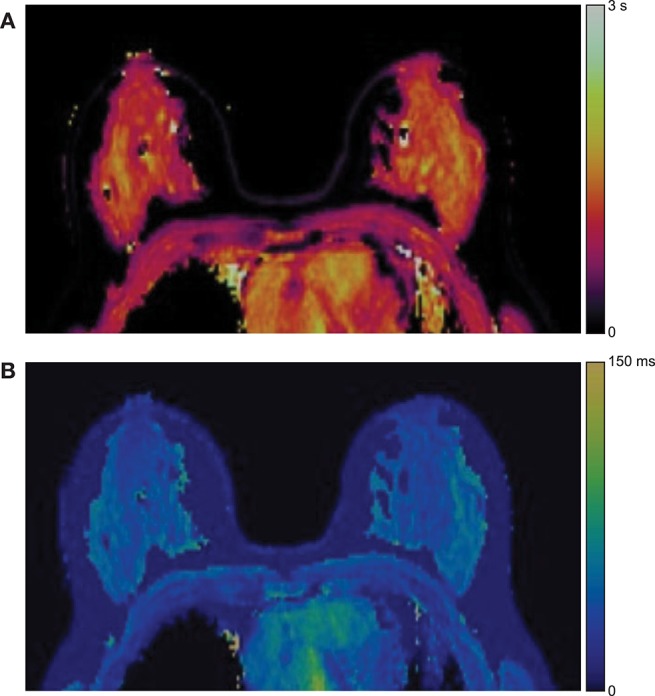
Example MR fingerprinting of the breast. Representative **(A)** T_1_ and **(B)** T_2_ MR fingerprinting color maps from one subject. Reprinted with permission from Chen et al. (109); ©2019 International Society for Magnetic Resonance in Medicine.

A relatively new technique known as MR fingerprinting utilizes a pseudorandom RF excitation and pattern recognition to produce quantitative maps of tissue properties (108). Results from preliminary breast MR fingerprinting studies illustrate the simultaneous quantitative mapping of T_1_ and T_2_ in a bilateral configuration (109, 110). Representative T_1_ and T_2_ MR fingerprinting maps are shown in Figure 7.

#### MR Electrical Properties Tomography

MR electrical properties tomography exploits typically undesirable distortions in the RF transmit field (B_1_) to reconstruct the conductivity and electrical permittivity of tissue (111). A preliminary breast MR electrical property tomography study by Shin et al. found malignant cancers have higher conductivity than benign lesions, and invasive cancers showed higher conductivity compared to DCIS (112).

#### Novel Contrast Agents

Recent discoveries of gadolinium retention within the body have raised questions regarding the long-term toxicity of gadolinium-based contrast agents and propelled the quest for novel contrast agents that are both safe and equally effective (113). Recent studies have begun reevaluating alternative contrast agents for breast cancer, including manganese (114, 115) and iron chelates (116). Even so, research continues on gadolinium-based contrast agent's improvements, and agents can be designed to target specific molecular peptides. A preclinical study utilized one such contrast agent to bind to fibrin-fibronectin complexes abundant in malignant cancer, including micro metastases (117). While human trials have not commenced, these novel contrast agents have potential to improve the early detection and characterization of high-risk breast tumors.

#### Machine Learning

Machine learning is a branch of data science that “trains” computers to learn data without preprograming the computers to perform specific tasks. There are two types of machine learning models: unsupervised learning and supervised learning. Unsupervised learning aims to classify data that have not been assigned labels or categories; examples include neural networks and clustering to map input data (e.g., breast images) into output categories that share similar contents (e.g., tumor assessments). On the other hand, supervised learning aims to classify data that have been assigned with ground truth labels (e.g., radiological assessments); example models include regression methods and support-vector machines (SVM).

As an artificial intelligence tool, machine learning may best be introduced to the clinic through structured use cases; in the case of breast cancer, these may include the application of artificial intelligence to identify suspicious microcalcifications (118) and, given the variability of visual density assessments (119), the quantification of breast fibroglandular tissue volume (25). The American college of radiology recommend using the BI-RADS categories for characterizing breast lesions. This method relies on the radiologist's experience and is limited by inter-observer variance.

Neural networks are machine learning models that consist of multiple interconnected layers. The study of neural networks is termed deep learning. Lately, deep learning has surpassed traditional image processing models in the segmentation and detection of novel imaging biomarkers (120). Convolutional neural networks are a type of neural network that has convolutional layers and hidden layers, and they have profound diagnostic performance. For example, a 3D deep convolution neural network can be used to identify and localize malignant breast lesions in DCE images, previously demonstrating 90.8% sensitivity and 69.3% specificity (121, 122). Another potential application is fibroglandular tissue and BPE assessment; while BI-RADS defines relevant categories, it does not establish percentage values for their quantification. A large proportion of fibroglandular tissue in the breast correlates with breast cancer risk (23, 26, 119, 123). Robust fibroglandular tissue quantification can be an efficient tool for clinicians to process large amount of breast MRI data and support more accurate breast cancer risk assessments (124). Independent of fibroglandular tissue quantification, computer-aided BPE quantification in DCE images has shown potential to be an imaging biomarker of breast cancer (125). For breast image segmentation and tumor volume quantification, several algorithmic routines have been demonstrated, e.g., (123, 124, 126–128); however, deep computational neural networks (i.e., U-nets) have shown particular promise for improving robustness and accuracy of results (129–131). Figure 2B shows the segmented fibroglandular tissue overlaid on anatomical DCE breast images. Based on fully automated computerized approaches, BPE DCE-MRI recently has been reported applicable in screening potential risk factors of breast cancer to regionalize the parenchymal tissues and their vasculature (125).

Radiomics involves extracting quantitative features from medical images, such as tumor size, shape, and textures, and patient-level data, such as the genetic data, to determine the underlying relationship between these features and pathologies (121, 132–136). A radiomics study of BPE DCE-MRI was able to differentiate subtypes of triple negative breast cancer (137). Another study combining BPE and T_2_-weighted breast MRI predicted NAC response with high accuracy (138). Texture parameters used as features in the support-vector machine learning approach show accurate prediction of benign and malignant breast lesions (133, 138–142). Texture parameters can consist of statistical and grey-level metrics in the sub-1cm region of interest in DCE images (139), the ADC map histogram combined with DCE-derived parametric maps (140, 141), and the parenchymal texture analysis (133). Finally, radiogenomics aims to identify imaging biomarkers and incorporates with phenotypic and genotypic metrics to support the execution of radiomics studies (142).

Machine learning has applications in breast lesion detection and classification, as well as predicting NAC response. Machine learning can bring together data from many studies and reduce the variability of radiologists' annotation methods on breast lesions. The current limitations of machine learning are the training requirement of large datasets and lack of standardized machine learning models to extract features from these datasets. Lastly, the decision-making process of machine learning can be considered a “black box”; it is difficult to intuitively explain how and why a certain answer is produced by machine learning models.

### Ultra-High Field MR Scanners

#### 7 Tesla

As indicated by the improved fat-water contrast visible in Figure 1, the positive predictive value and cancer detection rates of MRI increase at higher magnetic fields (143). However, the issue of transmit B_1_ inhomogeneity is greater at ultra-high fields, and it becomes necessary to utilize a local transmit coil for breast MRI at 7T (144). Given the proximity to the breasts and the greater net magnetization inherent at higher static magnetic fields, a local RF coil may be used for both transmit and receive (28). However, owing to the asymmetric dielectric load presented by the torso, transmit B_1_ inhomogeneity can still be pronounced throughout the breasts, leading to a linear signal drop-off toward the chest wall. In response, adiabatic pulse sequences have been developed to compensate for B_1_ inhomogeneity and improve tip angle uniformity (145). Alternatively, transmit coil designs exploiting transmission line techniques, e.g., forced current excitation (90, 146), have been shown to produce excellent B_1_ homogeneity throughout the breast to the chest wall [7.2% B_1_ coefficient of variation reported in (147)] and facilitate the use of standardized pulse sequences. As with lower static fields, the received SNR is further improved by utilizing a 7T array coil insert (148–151).

#### Ultra-High Field Safety

The potential for RF power deposition to cause localized tissue heating is more apparent at higher fields. The amount of power dissipated in a given mass of tissue is quantified as specific absorption rate, and operational safety limits are stipulated by the International Electrotechnical Commission (152). The safety of local transmit coils must be validated, typically through thermometry measurements and electromagnetic simulation of the specific coil design. While higher specific absorption rate is expected for women with greater breast tissue density, their resulting levels for routine 7T pulse sequences are generally well within safety limits (153, 154). Furthermore, a preliminary simulation study indicates the presence of breast implants has no significant effects on specific absorption rate or tissue heating (155).

## Conclusions And Future Directions

The current and emerging MRI techniques discussed in this paper are summarized in Table 1. For a multifaceted disease such as cancer, multi-parametric approach through which both structural and functional information can be elucidated simultaneously is a necessity to overcome the limitations of current MR based clinical modalities. In comparison to the stand-alone modalities, mpMRI enables both visualization and quantification. Quantifying varied cancer traits, including but not limited to, tumor architecture, tumor microenvironment, vascularization and angiogenesis, tumor heterogeneity, cellularity, metabolite concentration, and receptor status in parallel with image reconstruction through the combination of modalities would inevitably improve the status quo in detecting and treating breast cancer (156). Furthermore, individual modalities that appear far-removed from standalone efficacy may be ideal adjuncts for an mpMRI approach; for example, Weiss et al. recently demonstrated a promising approach to predict personalized response to NAC using a combination of DCE and DWI; however, the accuracy of their mathematical model would be strengthened by personalized measurements of elastic properties of the breast, potentially through MRE (157). Ultimately, mpMRI incorporating one or more emerging methods has the potential to afford improved specificity and deliver excellent accuracy for the prediction, detection, and monitoring of breast cancer (158).

**Table 1:** Comparison of current and emerging MRI techniques.

	**Imaging techniques**		**Clinical applications**	**Features and strengths**	**Limitations**
**Current MRI techniques**	Structural imaging		T_1_ and T_2_ weighted bilateral fat suppression imaging	Superior sensitivity for breast tumors; preferable for dense breast imaging	Low tumoral contrast, as tumor is surrounded by breast fat and fibroglandular tissue
	Contrast Enhanced Perfusion MRI	Dynamic Contrast Enhanced (DCE) MRI	Routinely utilized for distinguishing malignant vs benign cancers	Microvasculature and hypersensitivity in malignant tumors	Affected by hormones (menstrual cycle)
**Emerging MRI techniques**	Diffusion Weighted MRI (Gaussian)	Diffusion Weighted Imaging (DWI)	Potential tissue cellularity-based approach	Improved lesion detection for voxel-wise calculation (47, 48); higher resolution achievable (e.g., 0.8 mm) (47); yields superior quality when used in combination with MRS or other multiparametric modalities (46)	Inconsistency in obtaining high-quality breast DWI but can be solved with protocol standardization and QA procedure (see (49) for more details)
	Diffusion Weighted MRI (Non-Gaussian)	Diffusion Kurtosis Imaging	Potential to differentiate heterogenous tumor microstructures (62)	Applicable for intracellular structures, e.g., membranes and organelles (61); improved unsuppressed fat signal (63)	Low SNR; longer scanning time and higher magnetic gradient strength for high b-value acquisition
	Magnetic Resonance Spectroscopy (MRS)	Proton Spectroscopy	Potential biomarker for malignant breast cancer	Highest sensitivity and simplest data acquisition	Issues related to reproducibility across clinical sites (74)
	Other techniques	Sodium MRI	Potentially differentiating malignant tumors based on sodium concentration (101)	No chemical or spectral shift observed; based on sodium/potassium ion channels in the body	Could be overlapped with other sodium/potassium ion channel related disorder

Both DWI and ^1^H MRS are considered important approaches to pursue the analysis of tumor growth and treatment response *in vivo* (159). Advanced DWI methods that have the potential to distinguish tumors, given distinct signatures of cellularity and intravoxel heterogeneity, hold great potential in the noninvasive differentiation of tumor subtypes. Specifically, the fractional order calculus model (160) can derive micrometer-scale diffusion metrics that may reflect nuclear morphometry. To elicit sensitivity to shorter-scale diffusion, this method requires acquisitions with at least five b-values in the high range of b = 3000–4000 s/mm^2^. While one retrospective study failed to show improved utility of fractional order calculus model parameters as compared to DWI ADC, the maximum b-value acquisitions included in the study (b = 1500 s/mm^2^) were insufficient to properly evaluate the fractional order calculus model (161). Regarding ^1^H MRS, current issues surrounding inter-site reproducibility of single-voxel MRS may be mitigated through automated voxel placement or full 3D magnetic resonance spectroscopic imaging (74), particularly if following standardized process for acquisition, post-processing, and analysis (162). Continued development of MT techniques, including amide proton transfer, also show promise for differentiating tumor subtypes and predicting treatment outcome. DWI, MRS, and amide proton transfer all will benefit from the growing footprint of 7T MR scanners and continued progress toward U.S. Food and Drug Administration approval of clinical breast cancer applications at 7T. Positron emission tomography (PET) as a stand-alone imaging technique is known to have a high diagnostic ability for metastasis through imaging of the breast and adjacent lymph nodes. The diagnosis and characterization of primary tumors using PET has been shown to be improved when used simultaneously in conjugation with MRI, owing to the strengths of the individual modalities (163), but more research on combined PET/MRI modality is required to provide enough supportive evidence of their higher sensitivities. Radiation associated with the tracer in PET could be another concern; however, Melsaether et al. have demonstrated 50% reduction in total radiation dose when switching from PET/computed tomography to PET/MRI in a population of breast cancer patients, implying a safer mode of imaging and diagnosis in comparison to the former (164).

Finally, the rapidly advancing field of machine learning will facilitate more impactful applications for breast cancer detection and management, likely improving specificity, positive predictive value, and differentiation of tumor subtypes through MRI. Moreover, simultaneous assessments of biomarkers and their genomics data through radiogenomics is likely to prove instrumental in the future as we advance toward precision health or personalized medicine and simultaneously decrease the MRI associated false positive rates.

## Author Contributions

AC, XL, and JR drafted the manuscript. XL generated new figures. All authors contributed to manuscript revision and approved the submitted version.

## Conflict of Interest

The authors declare that the research was conducted in the absence of any commercial or financial relationships that could be construed as a potential conflict of interest.
